# A Synaptogenesis-Associated Histomorphologic Signature from H&E Whole-Slide Images Predicts Glioma Prognosis and Identifies *EFNB2*-Positive Malignant Cells as a Candidate Neuro-Glioma Communication Hub

**DOI:** 10.3390/ijms27104300

**Published:** 2026-05-12

**Authors:** Xiaolong Wu, Dong Liu, Haoming Geng, Binghan Zhang, Huantong Diao, Yiqiang Zhou, Gang Song, Ye Cheng, Jiantao Liang

**Affiliations:** 1 Department of Neurosurgery, Xuanwu Hospital, Capital Medical University, Beijing 100053, China; 2 China International Neuroscience Institute, Beijing 100053, China

**Keywords:** gliomas, neuron–glioma interactions, synaptogenesis, pathology, *EFNB2*

## Abstract

Synaptogenesis-related neuron–glioma interactions are increasingly recognized in glioma, yet it remains unclear whether routine H&E morphology can capture these programs and improve prognostic stratification. We integrated H&E whole-slide images, transcriptomes, and clinical data from 434 TCGA gliomas. Deep learning and quantitative pathology yielded an integrated histomorphologic feature set of 2678 features. Synaptogenesis-related activity was quantified using ssGSEA for ninety-eight synaptogenesis-related genes. In the training cohort, Spearman analysis identified 149 correlated histomorphologic features, which were refined to thirty-five by elastic net regularization. Seventeen prognostic candidates were entered into the MIME1 framework, and the most parsimonious model, Enet[0.1], retained fourteen non-zero-coefficient features to define the synaptogenesis-associated histomorphologic signature and construct the pathology-derived risk score (PRS). Multi-omic analyses, Human Protein Atlas validation, and single-nucleus RNA-seq were used to investigate the hub gene and its cellular context. PRS robustly stratified survival in both training and validation cohorts and remained an independent prognostic factor after adjustment for age and 2021 WHO CNS grade. High-risk tumors showed increased stromal and immune scores and enrichment of immune, adhesion, and phagosome-related pathways. *EFNB2* emerged as the hub gene and was enriched in glioblastoma, and *EFNB2*-positive malignant cells displayed prominent communication with neurons, including EFNB2-EPHB1 signaling. Exploratory re-analysis of the myeloid compartment further showed that glioblastoma was enriched for suppressive TAM-like states relative to astrocytoma grade 2, supporting a shift toward a more tumor-associated and potentially immunosuppressive microenvironment. Routine H&E histomorphology can capture synaptogenesis-related molecular programs in glioma. The resulting PRS provides clinically relevant prognostic stratification, while *EFNB2*-positive malignant cells may represent a candidate hub for neuron–tumor communication within a remodeled tumor ecosystem.

## 1. Introduction

Gliomas are among the most common primary tumors of the central nervous system, with glioblastoma (GBM) representing the most aggressive subtype, characterized by highly infiltrative growth, frequent recurrence, and dismal prognosis [1,2,3,4]. Although a standard multimodal treatment strategy based on maximal safe resection, radiotherapy, and temozolomide chemotherapy has been established, clinical outcomes remain markedly heterogeneous. Even among patients who receive comparable standard-of-care treatment and share the same integrated diagnosis under the 2021 World Health Organization Classification of Tumors of the Central Nervous System (WHO CNS), substantial differences can still be observed in treatment response, time to recurrence, and overall survival [1,4,5]. These observations suggest that conventional clinicopathological stratification is insufficient to fully explain the biological complexity of glioma, highlighting the urgent need for novel biomarkers that better reflect key tumor functional states and microenvironmental features.

The 2021 WHO CNS has substantially improved the accuracy and consistency of glioma classification by emphasizing integrated diagnosis based on both histological and molecular information [1]. Molecular markers such as *IDH* status, *1p/19q* codeletion, and TERT promoter mutation have become essential components of clinical decision-making [1]. However, in real-world practice, molecular testing remains constrained by cost, sample quality, platform variability, and regional accessibility. More importantly, significant prognostic heterogeneity persists even among patients with the same WHO CNS integrated diagnosis [4,5,6,7]. Therefore, there is clear clinical value in developing a novel stratification tool derived from routinely available clinical materials that can complement molecular classification while also reflecting the functional state of the tumor. With the rapid development of digital pathology and computational pathology, hematoxylin and eosin (H&E) whole-slide images (WSIs) are no longer merely substrates for morphological assessment, but are increasingly recognized as valuable data sources for extracting molecular features, microenvironmental characteristics, and prognostic information [8].

In recent years, cancer neuroscience has emerged as a rapidly evolving field, with accumulating evidence indicating that neurons are not merely passive targets of tumor growth, but can actively regulate tumor initiation, progression, and therapeutic response through multilevel and multiscale mechanisms [2,9,10]. Although neuron–glioma interaction has become a major focus of brain tumor research, most existing evidence has relied on transcriptomics, spatial omics, electrophysiological recordings, animal models, or complex in vitro co-culture systems [2,9,10,11,12,13,14]. A simple surrogate that can directly capture these neural interaction-related biological states from routine clinical pathology specimens is still lacking. In other words, a key unresolved question is whether the molecular states associated with neuron–glioma interaction, particularly those related to synaptogenesis and neuronal activity-dependent programs, leave quantifiable and reproducible histomorphologic footprints in routine H&E morphology. If so, computational histomorphologic features derived from WSIs may serve as a practical bridge linking tissue morphology, neural-related molecular programs, and clinical outcomes.

Based on this rationale, the present study integrated H&E WSIs, transcriptomic profiles, and survival outcomes from The cancer genome atlas (TCGA) glioma cohort to construct a pathology-derived risk score (PRS) centered on synaptogenesis-related gene sets. We first extracted both deep learning-derived features and interpretable quantitative histomorphologic features from routine WSIs, and then quantified synaptogenesis-related molecular activity using Gene Set Variation Analysis (GSVA)/single-sample gene set enrichment analysis (ssGSEA). Through correlation analysis, regularized feature selection, and survival modeling, we developed a synaptogenesis-associated histomorphologic signature capable of predicting glioma prognosis. Furthermore, by integrating differential expression analysis, tumor immune microenvironment assessment, Human Protein Atlas (HPA) immunohistochemical validation, and single-nucleus transcriptomic cell–cell communication analysis, we identified an *EFNB2*-related signaling network and explored the potential hub role of *EFNB2*-positive malignant cells in neuron–tumor communication.

Accordingly, this study focused on three major questions. First, can synaptogenesis-related molecular programs be effectively identified through computational histomorphologic features derived from routine H&E slides? Second, can this synaptogenesis-associated histomorphologic signature provide additional prognostic stratification beyond current WHO-related classification frameworks? Third, what are the key genes and intercellular communication networks underlying this morphology–molecular coupling, and do they point to neuron–tumor interaction axes that merit further mechanistic validation and potential therapeutic targeting?

## 2. Results

### 2.1. Synaptogenesis-Associated Histomorphologic Features

**Figure 1:**
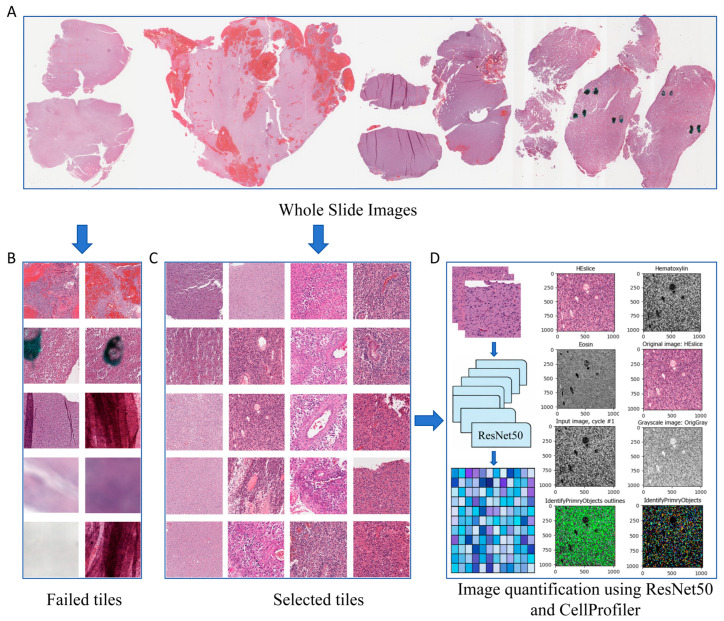
Workflow for whole-slide image processing and construction of the integrated histomorphologic feature set. (**A**) Representative H&E-stained WSIs from glioma samples. (**B**) Examples of excluded low-quality tiles, including tiles with artifacts, blur, handwriting marks, tissue folds, staining interference, or insufficient informative tissue content. (**C**) Representative high-quality tiles retained for downstream analysis after quality control. (**D**) Schematic overview of histopathological image quantification. Deep learning features were extracted from histopathological tiles using a pretrained ResNet50 network, generating 2048-dimensional feature vectors, whereas CellProfiler was used to extract 630 quantitative histomorphologic features from H&E images and stain-separated grayscale channels. Together, these features constituted an integrated histomorphologic feature set for subsequent analyses. H&E, Hematoxylin and eosin; WSIs, whole-slide images.

A total of 434 glioma cases with matched histopathological slides, transcriptomic profiles, and clinical follow-up data were ultimately included for model development and downstream analyses. Based on a comprehensive review of previously published studies, ninety-eight synaptogenesis-related genes (Supplementary Table S1) were compiled to represent synaptogenesis-related molecular activity in glioma. For histopathological image feature extraction (Figure 1), we first used a pretrained ResNet50 network to encode each histopathological image tile, generating a 2048-dimensional feature vector for each tile. These tile-level deep learning features were then aggregated to the case level by averaging across all tiles from the same whole-slide image, thereby capturing high-dimensional information related to tissue architecture, cellular organization, and morphological patterns. In parallel, we applied CellProfiler (v4.2.4) to extract 630 quantitative histomorphologic features, including measurements related to staining intensity, texture, granularity, area, shape, and nuclear morphology. For each case, the mean, median, and standard deviation of each CellProfiler-derived feature across all tiles were calculated to summarize both the overall level and spatial heterogeneity of the histomorphologic features. Together, the 2048 ResNet50-derived deep learning features and 630 CellProfiler-derived quantitative histomorphologic features constituted an integrated histomorphologic feature set of 2678 features.

### 2.2. Selection of Synaptogenesis-Associated Histomorphologic Features and Construction of the Synaptogenesis-Associated Histomorphologic Signature

**Figure 2:**
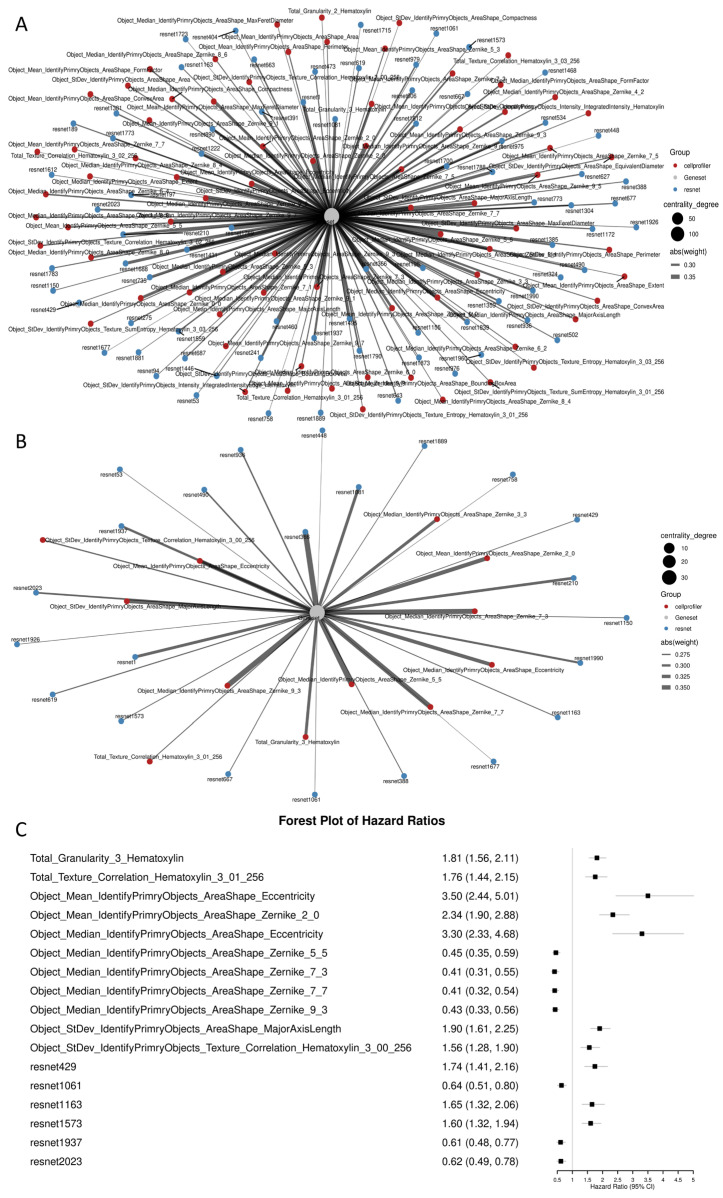
Selection of synaptogenesis-associated histomorphologic features associated with prognosis. (**A**) Correlation network between synaptogenesis-related gene scores and histomorphologic features in the training cohort. A total of 149 histomorphologic features significantly associated with synaptogenesis-related gene scores were identified by Spearman correlation analysis. Red nodes indicate CellProfiler-derived quantitative histomorphologic features, blue nodes indicate ResNet50-derived deep learning features, node size reflects centrality, and edge width indicates the absolute correlation strength. (**B**) Refined correlation network after elastic net regularization (α = 0.2), showing thirty-five robust and non-redundant histomorphologic features related with synaptogenesis-related gene scores. (**C**) Forest plot of univariate Cox regression for the seventeen prognostic histomorphologic features selected from the candidate set. Hazard ratios (HRs) and 95% confidence intervals (CIs) are shown.

All samples were randomly divided into a training set and a validation set at a ratio of 3:1. In the training cohort, Spearman correlation analysis identified 149 histomorphologic features that were significantly associated with synaptogenesis-related gene scores (Figure 2A). These candidate features were then further refined using elastic net regression (α = 0.2) to reduce dimensionality and eliminate redundancy, resulting in thirty-five histomorphologic features stably associated with synaptogenesis-related factors (Figure 2B). Univariate Cox regression was subsequently performed on all candidate features, and seventeen histomorphologic features significantly associated with overall survival (OS) were ultimately retained for model construction (Figure 2C).

**Figure 3:**
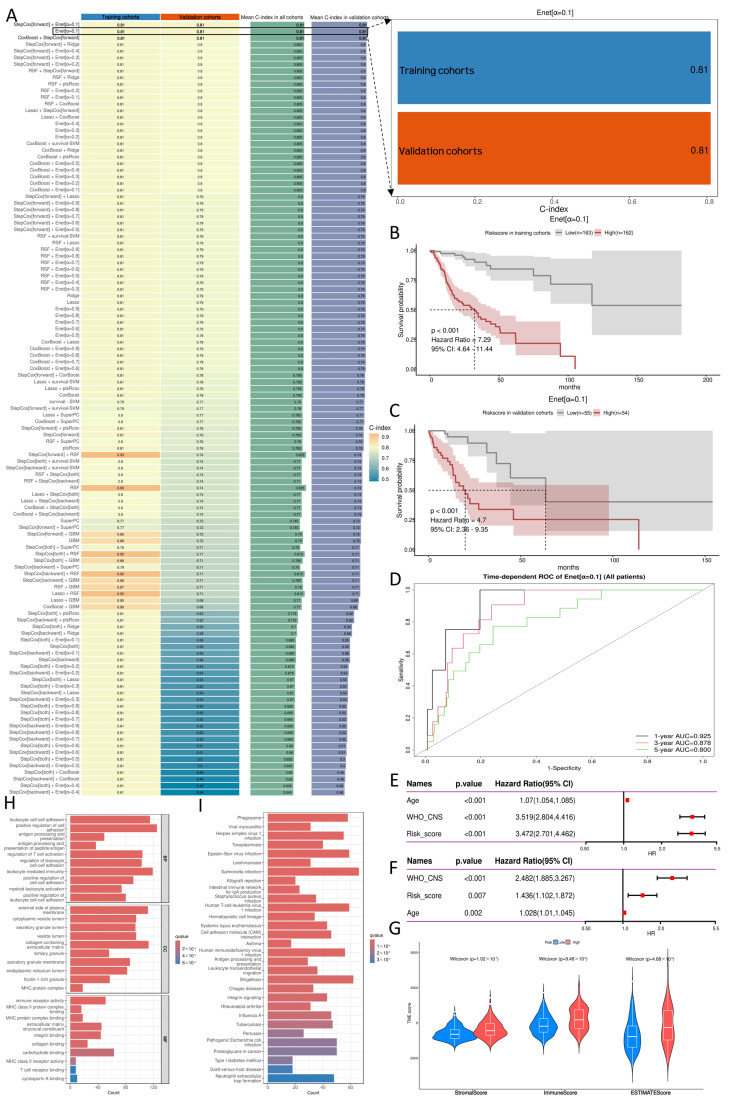
Construction and validation of the synaptogenesis-associated histomorphologic signature and PRS. (**A**) Performance comparison of 100 machine learning algorithms and their combinations within the MIME1 framework. Model performance was evaluated using C-index in the training and validation cohorts. Among the top-performing models, Enet[0.1] was selected as the final model because of its comparable predictive performance and greater parsimony. Inspection of the Enet[0.1] coefficients showed that fourteen of the seventeen candidate histomorphologic features retained non-zero coefficients and were therefore used to define the final synaptogenesis-associated histomorphologic signature and establish the PRS. (**B**) Kaplan–Meier overall survival analysis of the high- and low-risk groups in the training cohort, stratified by PRS. (**C**) Kaplan–Meier overall survival analysis of the high- and low-risk groups in the validation cohort, stratified by PRS. (**D**) Time-dependent ROC curves of the final model for predicting 1-, 3-, and 5-year overall survival in the entire cohort. (**E**) Univariate Cox regression analysis including age, 2021 WHO CNS grade, and PRS. (**F**) Multivariate Cox regression analysis showing that PRS remained an independent prognostic factor after adjustment for age and 2021 WHO CNS grade. (**G**) Comparison of StromalScore, ImmuneScore, and ESTIMATEScore between the low- and high-risk groups defined by PRS. (**H**) GO enrichment analysis of differentially expressed genes between the two PRS-defined risk groups, including BP, CC, and MF categories. (**I**) KEGG pathway enrichment analysis of differentially expressed genes between the low- and high-risk groups defined by PRS. PRS, pathology-derived risk score; C-index, Harrell’s concordance index; BP, Biological Process; CC, Cellular Component; MF, Molecular Function; KEGG, Kyoto Encyclopedia of Genes and Genomes; GO, Gene Ontology.

Based on these seventeen histomorphologic features, prognostic models were developed within the MIME1 framework by evaluating 100 machine learning algorithms and their combinations, including StepCox, Lasso, Ridge, partial least squares Cox regression (plsRcox), CoxBoost, random survival forest (RSF), gradient boosting machine (GBM), elastic net (Enet), SuperPC, and survival support vector machine (survival-SVM) (Figure 3A). Model performance was assessed using Harrell’s concordance index (C-index) in both the training and validation cohorts. StepCox[forward] + Enet[0.1], Enet[0.1], and CoxBoost + StepCox[forward] achieved identical C-index values in both cohorts. Therefore, given comparable predictive performance, the most parsimonious model, Enet[0.1], was selected as the final model (Figure 3A). Further inspection of the Enet[0.1] model coefficients showed that, among the seventeen candidate histomorphologic features, three had coefficients shrunk to zero, whereas fourteen retained non-zero coefficients (Supplementary Table S3), including ten CellProfiler-derived quantitative histomorphologic features and four ResNet50-derived deep learning features. To improve interpretability, the quantitative definitions and putative morphologic relevance of the retained handcrafted features are summarized in Supplementary Table S3. These fourteen retained histomorphologic features were therefore used to define the final synaptogenesis-associated histomorphologic signature and to establish the PRS.

Kaplan–Meier survival analysis showed that patients in the high-risk group had significantly shorter overall survival than those in the low-risk group in both the training cohort (*p* < 0.001, HR = 7.29) and the validation cohort (*p* < 0.001, HR = 4.70) (Figure 3B,C). Time-dependent Receiver Operating Characteristic (ROC) analysis in the entire cohort further demonstrated the robust predictive performance of this model for 1-, 3-, and 5-year survival, with area under the curve (AUC) values of 0.925, 0.878, and 0.800, respectively (Figure 3D). Collectively, these findings indicate that the PRS, derived from the synaptogenesis-associated histomorphologic signature, has strong prognostic stratification ability and good robustness.

### 2.3. Further Evaluation of the Pathology-Derived Risk Score

To further assess the independent prognostic value of PRS, 2021 WHO CNS grade, age, and PRS were included in both univariate and multivariate Cox proportional hazards models. In the univariate analysis, all three variables—2021 WHO CNS grade, age, and PRS—were significantly associated with overall survival. Importantly, multivariate Cox regression demonstrated that PRS remained statistically significant after adjustment for 2021 WHO CNS grade and age, indicating that it served as an independent prognostic factor. Specifically, PRS yielded a hazard ratio (HR) of 3.472 (95% CI, 2.701–4.462) in the univariate Cox analysis and an HR of 1.436 (95% CI, 1.102–1.872) in the multivariate Cox analysis (Figure 3E,F). The PRS could be viewed as complementary to, rather than a substitute for, WHO CNS grade, because it captures additional histomorphologic risk information associated with synaptogenesis-related biological activity.

### 2.4. Association of PRS with the Tumor Microenvironment and Biological Functions

As shown in Figure 3G, the StromalScore, ImmuneScore, and ESTIMATEScore were all significantly higher in the high-risk group than in the low-risk group, indicating that increasing PRS was associated with greater stromal and immune infiltration and a more prominent non-tumor microenvironmental component. Accordingly, PRS was likely inversely associated with inferred tumor purity, suggesting that high-risk tumors not only exhibited a more aggressive histomorphologic phenotype but were also accompanied by more extensive microenvironmental remodeling.

To further investigate the biological differences between the two risk groups, we performed Gene Ontology (GO) and Kyoto Encyclopedia of Genes and Genomes (KEGG) enrichment analyses on the differentially expressed genes. GO analysis revealed that these genes were predominantly enriched in immune regulation- and cell adhesion-related processes. At the level of Biological Process (BP), the most significantly enriched terms included leukocyte cell–cell adhesion, positive regulation of cell adhesion, antigen processing and presentation of peptide antigen, regulation of leukocyte cell–cell adhesion, and myeloid leukocyte activation. In the Cellular Component (CC) category, the enriched terms were mainly associated with the external side of plasma membrane, cytoplasmic vesicle lumen, secretory granule lumen, vesicle lumen, collagen-containing extracellular matrix, and MHC protein complex. In the Molecular Function (MF) category, the dominant terms included immune receptor activity, MHC class II protein complex binding, MHC protein complex binding, integrin binding, and collagen binding (Figure 3H). Together, these findings suggest that high-risk tumors are characterized by enhanced immune activation, antigen presentation, cell adhesion, and extracellular matrix remodeling.

KEGG pathway analysis further showed that the differentially expressed genes were mainly enriched in immune- and inflammation-related pathways, including Phagosome, Antigen processing and presentation, Leukocyte transendothelial migration, Cell adhesion molecules (CAMs), and Hematopoietic cell lineage, as well as several infection- and immune response-associated pathways, such as Staphylococcus aureus infection, Herpes simplex virus 1 infection, Human T-cell leukemia virus 1 infection, Systemic lupus erythematosus, and Allograft rejection (Figure 3I). Overall, these enrichment results indicate that the high-risk group is associated not only with adverse histomorphologic features but also with a more active immune-inflammatory state, enhanced antigen processing and presentation, increased cell adhesion, and extensive microenvironmental remodeling.

### 2.5. Multi-Omic Characterization of the Hub Gene

**Figure 4:**
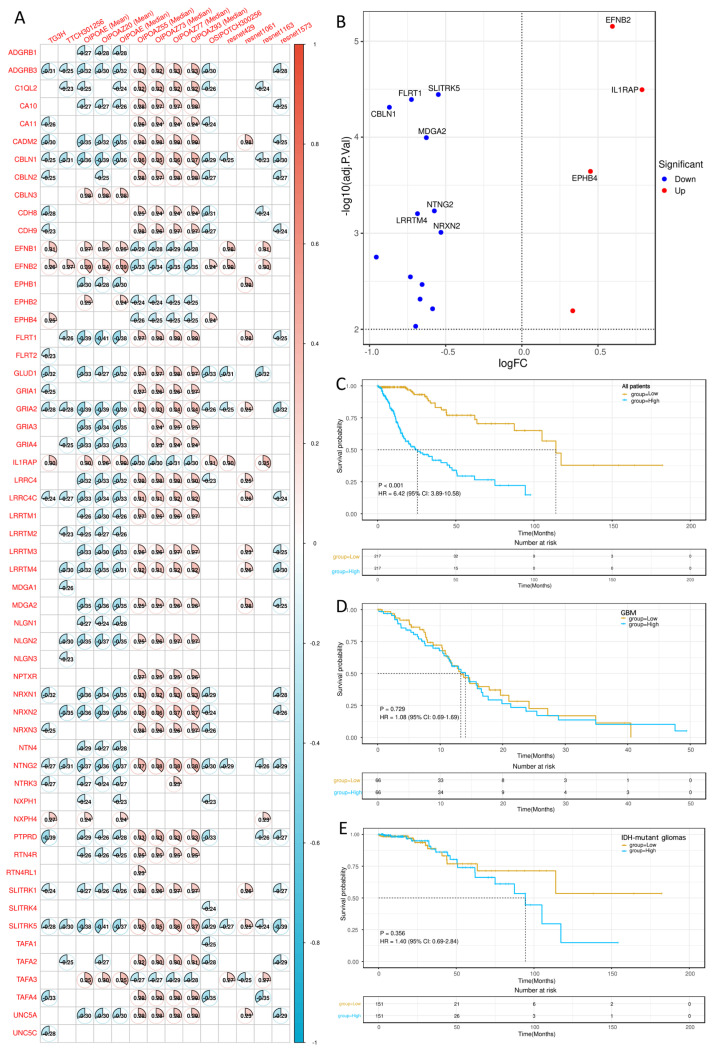
Multi-omic identification of *EFNB2* as the hub gene. (**A**) Correlation matrix between the fourteen histomorphologic features constituting the final synaptogenesis-associated histomorphologic signature and synaptogenesis-related genes. Circle color indicates the direction of correlation, and circle size reflects correlation strength. (**B**) Volcano plot showing the overlapping genes between transcriptome-wide differentially expressed genes identified from the high- versus low-PRS comparison and the fifty-six synaptogenesis-related genes associated with the histomorphologic feature–gene network. *EFNB2* showed the strongest statistical significance among the overlapping candidates and was therefore selected as the hub gene. (**C**) Kaplan–Meier overall survival analysis of all patients stratified by *EFNB2* expression level. (**D**) Kaplan–Meier overall survival analysis of GBM patients stratified by *EFNB2* expression level. (**E**) Kaplan–Meier overall survival analysis of *IDH*-mutant glioma patients stratified by *EFNB2* expression level.

To investigate the molecular basis underlying the key histomorphologic features, we further correlated the fourteen retained histomorphologic features included in the final synaptogenesis-associated histomorphologic signature with transcriptomic profiles from TCGA glioma samples and constructed a histomorphologic feature–synaptogenesis-related gene network (Figure 4A). This analysis showed that the fourteen model-included histomorphologic features were significantly associated with fifty-six synaptogenesis-related genes, suggesting that these genes may contribute to the histomorphologic alterations linked to patient prognosis. We first performed differential expression analysis between the high- and low-risk groups at the transcriptome-wide level. The resulting differentially expressed genes were then intersected with the fifty-six synaptogenesis-related genes identified from the histomorphologic feature–gene correlation analysis, and the overlapping genes are shown in the volcano plot in Figure 4B. Among these overlapping candidates, *EFNB2* showed the strongest statistical significance and was therefore selected as the hub gene for further analysis. Kaplan–Meier analysis in the entire cohort further demonstrated that high *EFNB2* expression was significantly associated with unfavorable overall survival, with patients in the high-expression group showing markedly worse outcomes than those in the low-expression group (Figure 4C). However, this association was no longer observed after subgroup stratification. When the entire cohort was divided into a GBM subgroup and an *IDH*-mutant glioma subgroup, Kaplan–Meier analysis showed no significant survival difference between *EFNB2*-high and *EFNB2*-low cases in either subgroup (Figure 4D,E). These findings suggest that the apparent adverse prognostic effect of *EFNB2* in the overall cohort is likely driven, at least in part, by its uneven distribution across WHO-integrated glioma subtypes rather than by a subtype-independent effect. Therefore, *EFNB2* may be more reflective of a subtype-enriched malignant biological state than a universal prognostic determinant across all glioma categories.

**Figure 5:**
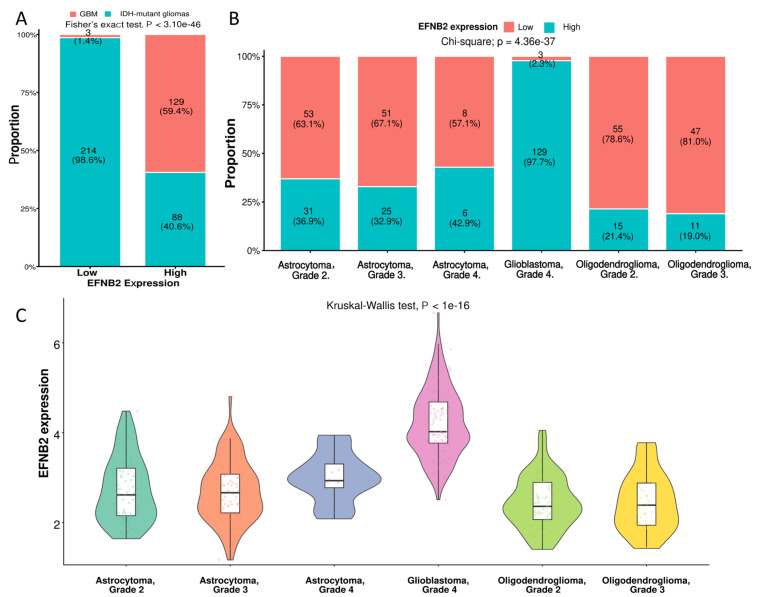

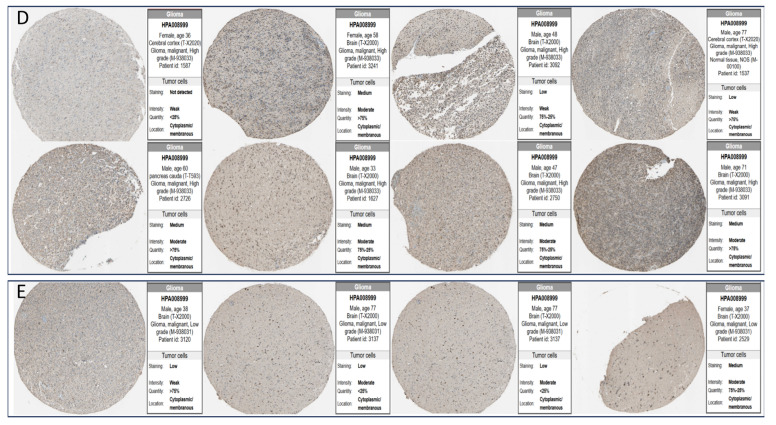
Distribution and validation of *EFNB2* expression across glioma subtypes and grades. (**A**) Distribution of GBM and *IDH*-mutant gliomas in the *EFNB2*-low and *EFNB2*-high groups. Samples were dichotomized by the median *EFNB2* expression level. Fisher’s exact test was used for comparison. (**B**) Proportions of *EFNB2*-low and *EFNB2*-high cases across six 2021 WHO CNS glioma categories. The chi-square test was used to compare subtype distributions. (**C**) Violin plot showing continuous *EFNB2* expression levels across six glioma categories, with the highest expression observed in glioblastoma, grade 4. The Kruskal–Wallis test was used for overall comparison. (**D**) Representative immunohistochemical staining images from the HPA showing *EFNB2* expression in high-grade glioma samples. (**E**) Representative HPA immunohistochemical staining images showing *EFNB2* expression in low-grade glioma samples.

To further define the expression landscape of *EFNB2* in glioma, all samples were dichotomized into *EFNB2*-high and *EFNB2*-low groups using the median expression level as the cutoff, and the proportions of GBM and *IDH*-mutant glioma cases were compared between the two groups. GBM was markedly enriched in the *EFNB2*-high group: in the *EFNB2*-low group, GBM accounted for only 1.4% (3/217) of cases, whereas in the EFNB2-high group, the proportion of GBM increased to 59.4% (129/217) (Figure 5A). This result indicates that elevated *EFNB2* expression is strongly associated with the GBM phenotype. We next examined *EFNB2* expression across the six WHO-based glioma categories, including astrocytoma grade 2, astrocytoma grade 3, astrocytoma grade 4, glioblastoma grade 4, oligodendroglioma grade 2, and oligodendroglioma grade 3. Notably, GBM was almost entirely classified as *EFNB2*-high, with 97.7% (129/132) of GBM samples belonging to the *EFNB2*-high group, whereas the other glioma subtypes were predominantly *EFNB2*-low (Figure 5B). Consistently, comparison of continuous *EFNB2* expression across the six WHO categories revealed a highly significant overall difference, with *IDH*-wildtype GBM showing the highest *EFNB2* expression, whereas *IDH*-mutant astrocytomas and oligodendrogliomas generally exhibited lower levels (Figure 5C). Together, these findings suggest that *EFNB2* is more likely to reflect a GBM-associated malignant biological state than a universal prognostic marker across all glioma subtypes. Transcriptomic analysis based on TCGA data showed that *EFNB2* was significantly upregulated in GBM compared with *IDH*-mutant gliomas. To further validate this observation at the protein level, we examined immunohistochemical staining data from the HPA. Representative images suggested that, compared with low-grade gliomas, high-grade gliomas displayed stronger *EFNB2* staining intensity and/or a higher fraction of positive tumor cells (Figure 5D,E), which was broadly consistent with the transcriptomic findings. These results further support a close association between *EFNB2* and a more aggressive, high-grade glioma phenotype.

**Figure 6:**
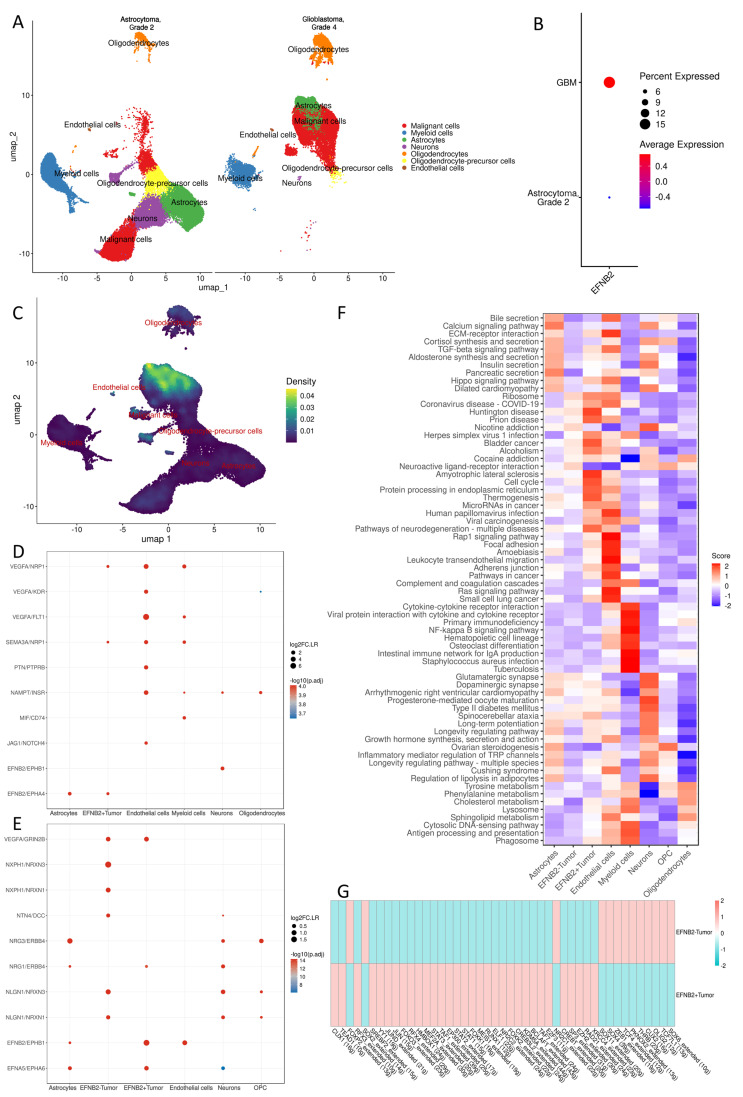
Single-nucleus transcriptomic characterization of *EFNB2*-associated malignant cells and neuron–tumor communication. (**A**) Integrated UMAP visualization of snRNA-seq data from astrocytoma, grade 2 and glioblastoma, grade 4, showing the major annotated cell populations, including malignant cells, myeloid cells, astrocytes, neurons, oligodendrocytes, oligodendrocyte precursor cells (OPCs), and endothelial cells. (**B**) Dot plot comparing *EFNB2* expression between astrocytoma, grade 2 and glioblastoma, grade 4. Dot size indicates the proportion of expressing cells, and color indicates average expression level. (**C**) UMAP feature-density plot showing the distribution of *EFNB2* expression across cell populations. (**D**) Bubble plot of representative significant ligand–receptor interactions centered on *EFNB2*-positive malignant cells under one communication direction setting, highlighting *EFNB2*-related signaling, including EFNB2–EPHB1 interactions. (**E**) Bubble plot of representative significant ligand–receptor interactions centered on neurons under the reciprocal communication setting, again showing EFNB2–EPHB1 and additional neuron-related signaling pairs. Dot size represents interaction strength, and color reflects statistical significance. (**F**) Heatmap of KEGG pathway activity quantified by GSVA across major cell populations, showing distinct functional programs in *EFNB2*-positive malignant cells, *EFNB2*-negative malignant cells, and neurons. (**G**) Heatmap of regulon activity inferred by SCENIC comparing *EFNB2*-positive and *EFNB2*-negative malignant cells, illustrating differences in transcription factor regulatory programs between the two malignant cell states.

To further explore the cellular distribution and potential function of *EFNB2* across glioma grades, we integrated two independent glioma single-nucleus transcriptomic datasets, including four astrocytoma grade 2 samples and six GBM samples (Supplementary Table S2). After stringent quality control and initial clustering, a total of 40,949 cells were retained for downstream analysis. Based on canonical marker genes, seven major cell populations were annotated, including malignant cells, myeloid cells, astrocytes, neurons, oligodendrocytes, oligodendrocyte precursor cells (OPCs), and endothelial cells (Figure 6A). Expression analysis showed that *EFNB2* expression was higher in GBM than in astrocytoma grade 2 (Figure 6B), and was mainly detected in malignant cells, astrocytes, neurons, and endothelial cells (Figure 6C). Cell–cell communication analysis further revealed that, within the functionally filtered communication network, *EFNB2*-positive malignant cells displayed the highest node strength, indicating the greatest total interaction number and interaction strength (Supplementary Figure S1). This suggests that *EFNB2*-positive malignant cells occupy a central position in the tumor microenvironmental communication network and may actively exchange ligand–receptor signals with myeloid cells, astrocytes, neurons, endothelial cells, and themselves. More detailed ligand–receptor analyses further delineated the complex signaling relationships between *EFNB2*-positive malignant cells and other cell types. Notably, when *EFNB2*-positive malignant cells and neurons were each considered as upstream or downstream clusters, the EFNB2/EPHB1 interaction axis was identified in both directions (Figure 6D,E), suggesting bidirectional Ephrin–Eph signaling between these two cell populations. In addition to EFNB2/EPHB1, the interaction maps also highlighted several signaling pairs related to neural development and synaptic function, including NLGN1/NRXN1, NLGN1/NRXN3, NRG1/ERBB4, NRG3/ERBB4, NXPH1/NRXN1, NXPH1/NRXN3, and NTN4/DCC, further supporting a neuron-like or synapse-associated mode of communication between *EFNB2*-positive malignant cells and neurons. To compare the functional states of different cell populations, significant ligand–receptor interactions were mapped to KEGG pathways, and pathway activity was quantified using GSVA (Figure 6F). Clear cell-type-specific patterns were observed. Compared with *EFNB2*-negative malignant cells, *EFNB2*-positive malignant cells showed stronger enrichment in pathways related to cell cycle, protein processing in endoplasmic reticulum, thermogenesis, microRNAs in cancer, viral carcinogenesis, Rap1 signaling pathway, focal adhesion, adherens junction, complement and coagulation cascades, Ras signaling pathway, and pathways in cancer, suggesting a more proliferative, adhesive, migratory, and microenvironment-remodeling phenotype. By contrast, *EFNB2*-negative malignant cells showed overall weaker pathway activity across these tumor-associated programs. On the other hand, neurons were preferentially enriched in pathways such as glutamatergic synapse, dopaminergic synapse, long-term potentiation, and inflammatory mediator regulation of TRP channels, indicating that neurons mainly contributed synaptic and neuroactive signaling inputs, whereas *EFNB2*-positive malignant cells were more likely to translate such external neuronal cues into pro-tumor programs related to growth and invasion. Finally, we performed SCENIC analysis to infer transcription factor regulatory networks in *EFNB2*-positive and *EFNB2*-negative malignant cells (Figure 6G). Importantly, this heatmap represents differences in regulon activity, rather than differences in average gene expression. The analysis revealed widespread remodeling of transcriptional regulatory programs between the two malignant cell states. Compared with *EFNB2*-negative malignant cells, *EFNB2*-positive malignant cells showed higher activity of several regulons, including JUN, JUND, FOXO3, STAT3, STAT2, STAT1, EP300, RUNX1, and ELK4, whereas other regulons, such as SOX4, SOX11, ZNF263, TCF4, and PBX3, were relatively more active in *EFNB2*-negative malignant cells. These findings suggest that *EFNB2*-positive malignant cells may be driven by a distinct set of inflammatory, stress-adaptive, and tumor-associated transcriptional programs, which could underlie their more active signaling interactions with neurons and other components of the tumor microenvironment.

### 2.6. Re-Annotation of the Myeloid Compartment Reveals Enrichment of Suppressive TAM-like States in GBM

**Figure 7:**
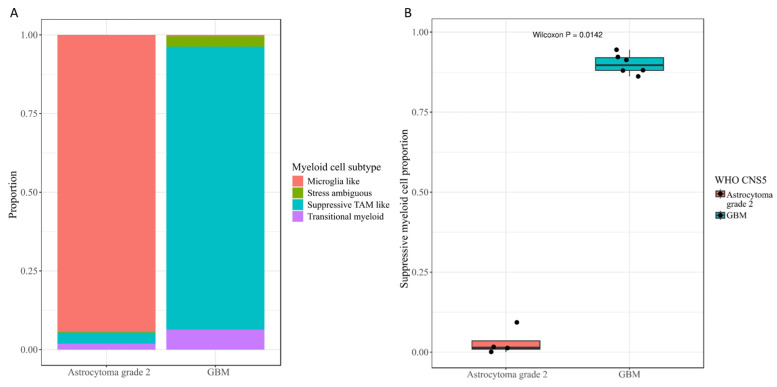
Myeloid remodeling toward suppressive TAM-like states in glioblastoma. (**A**) Composition of re-annotated myeloid states in astrocytoma grade 2 and glioblastoma (GBM), showing a predominance of Microglia-like cells in astrocytoma grade 2 and marked enrichment of Suppressive TAM-like cells in GBM. (**B**) Sample-level comparison of suppressive myeloid cell proportions within the myeloid compartment between astrocytoma grade 2 and GBM. GBM showed a significantly higher proportion of suppressive myeloid cells than astrocytoma grade 2 (Wilcoxon *p* = 0.0142), supporting a shift toward a more tumor-associated and potentially immunosuppressive myeloid microenvironment during glioma progression.

To further investigate whether the immune-enriched state inferred from bulk transcriptomic analysis was accompanied by specific myeloid remodeling, we performed an exploratory re-analysis of the myeloid compartment in the integrated single-nucleus transcriptomic dataset. Myeloid cells were re-clustered and annotated into four major states, including Microglia-like, Suppressive tumor-associated macrophage (TAM)-like, Transitional myeloid, and Stress-ambiguous populations, based on their clustering pattern, canonical marker expression, and program scores (Supplementary Figure S2A,B). Among these states, the Microglia-like population was characterized by homeostatic microglial markers, whereas the Suppressive TAM-like population showed stronger expression of tumor-associated and suppressive myeloid features. When stratified by glioma subtype, the myeloid landscape differed markedly between astrocytoma grade 2 and GBM. The astrocytoma grade 2 samples were dominated by Microglia-like cells, whereas GBM samples were markedly enriched for Suppressive TAM-like cells (Supplementary Figure S2C and Figure 7A). At the sample level, the proportion of suppressive myeloid cells within the myeloid compartment was significantly higher in GBM than in astrocytoma grade 2 (Wilcoxon *p* = 0.0142; Figure 7B), supporting a shift from a more homeostatic microglia-like state toward a more tumor-associated and potentially immunosuppressive myeloid state during glioma progression. We further explored whether a similar pattern could be observed in the *EFNB2*-associated malignant context. Samples with higher malignant-cell *EFNB2* expression showed a similar directional trend toward increased suppressive myeloid proportions; however, this difference did not reach statistical significance (Wilcoxon *p* = 0.144; Supplementary Figure S3). These findings provide exploratory single-cell-level support for the interpretation that the immune-enriched state observed in high-risk tumors is accompanied by myeloid remodeling toward suppressive TAM-like programs, while also indicating that the current sample size is insufficient for definitive subgroup-level conclusions regarding *EFNB2* context.

## 3. Discussion

The present study addressed a central question: whether routine H&E histomorphology can capture synaptogenesis-related molecular programs and, in turn, be leveraged for prognostic stratification and mechanistic discovery in glioma. To this end, we integrated TCGA whole-slide images, bulk RNA-seq data, and clinical follow-up information, and further incorporated immunohistochemical validation from the Human Protein Atlas as well as two independent snRNA-seq datasets. By doing so, we established a multilayered analytical framework linking digital histomorphologic features, transcriptomic programs, tumor microenvironmental states, and cell–cell communication mechanisms. Within this framework, our main findings were threefold. First, computational histomorphologic features extracted from routine H&E slides were able to effectively capture synaptogenesis-related molecular activity. Second, the PRS model provided additional prognostic stratification beyond established clinicopathological variables. Third, multi-omic integration and single-nucleus analyses identified *EFNB2* as a key candidate linking histomorphology, risk status, and neuron–tumor interaction, while *EFNB2*-positive malignant cells emerged as a putative communication hub within the glioma microenvironment.

Our findings further support the increasingly recognized concept that key molecular programs can leave quantifiable phenotypic projections in routine histopathology. The 2021 WHO CNS reinforced the central role of molecular information in glioma diagnosis while still preserving histology as a foundational layer of classification, underscoring that morphology and molecular biology are complementary rather than mutually exclusive [1]. Recent advances in computational pathology have shown that H&E images contain rich biological information beyond morphology alone. Deep learning approaches have already demonstrated that histology images can be combined with genomic features to improve survival prediction in brain tumors [15], and more recent studies have shown that spatial cellular architecture and localized digital histomorphologic phenotypes can reflect transcriptomic subtypes and clinical outcomes in glioblastoma [16,17]. In contrast to studies that directly model survival or single molecular alterations, our approach used a synaptogenesis-related factor gene set as a functional anchor and then traced back to the histomorphologic features most strongly linked to that program. This strategy not only yielded a prognostic model but also enhanced biological interpretability by connecting morphological signals to a defined neural-related molecular program.

The biological rationale for this work is closely aligned with the rapidly expanding field of cancer neuroscience. Neurons are not merely passive bystanders in the presence of brain tumors, but active regulators of tumor initiation, progression, and treatment response [18]. In glioma, neuronal activity promotes high-grade glioma growth through activity-dependent release of neuroligin-3 (NLGN3), which activates tumor-promoting pathways including PI3K–mTOR signaling [19]. High-grade glioma cells can form bona fide AMPAR-dependent neuron-to-glioma synapses, directly converting neuronal electrical activity into depolarization and proliferative signaling within tumor cells [14]. Glutamatergic synaptic input itself is sufficient to drive glioma progression [13]. In parallel, tumor microtubes (TMs) and tumor cell networks have been identified as important structural substrates of glioma invasion, growth, and therapy resistance [20]. More recently, human studies have shown that glioblastoma can remodel neural circuits, and that stronger tumor–brain functional connectivity is associated with worse survival and greater cognitive impairment [21], whereas BDNF-TrkB signaling further promotes malignant synaptic plasticity and tumor progression [22]. Within this conceptual framework, the PRS established here is unlikely to represent a purely morphological risk pattern. It more likely captures the integrated histomorphologic output of neuronal activity-dependent tumor programs, including synapse-like integration, networked tumor behavior, and microenvironmental remodeling.

Another important finding of this study is that high-PRS tumors exhibited significantly higher StromalScore, ImmuneScore, and ESTIMATEScore, together with enrichment of pathways such as phagosome, antigen processing and presentation, cell adhesion molecules, and leukocyte transendothelial migration. This should not be interpreted as evidence of a more effective anti-tumor immune response. Instead, these results likely reflect a more complex, immune-enriched, but potentially immunosuppressive and stromally remodeled microenvironment. Recent reviews have emphasized that glioblastoma is embedded within a highly dynamic ecosystem composed of neurons, astrocytes, oligodendrocyte-lineage cells, endothelial cells, immune cells, and extracellular matrix components, all of which can be reprogrammed by tumor cells to support growth, invasion, and treatment resistance [23]. Therefore, the increased immune and stromal scores observed in the high-risk group likely indicate not enhanced anti-tumor immunity, but rather a more inflammatory, more remodeled, and potentially more immune-suppressive microenvironmental state, which is consistent with the extensive interactions we observed between *EFNB2*-positive malignant cells and myeloid, endothelial, and astrocytic populations at the single-nucleus level. Our additional myeloid-focused single-nucleus analysis provides preliminary cellular support for this interpretation. After re-annotation of the myeloid compartment, astrocytoma grade 2 samples were found to be dominated by Microglia-like cells, whereas glioblastoma samples were markedly enriched for operationally defined Suppressive TAM-like cells. At the sample level, the suppressive myeloid proportion was significantly higher in GBM than in astrocytoma grade 2 (Wilcoxon *p* = 0.0142), whereas the *EFNB2*-context analysis showed only a non-significant trend toward higher suppressive myeloid proportions in *EFNB2*-high malignant contexts (Wilcoxon *p* = 0.144). Together, these findings suggest that the biological significance of our pathology-derived risk score is not confined to malignant cells alone, but extends to a dynamically remodeled tumor ecosystem that includes distinct myeloid states. Future studies integrating larger single-cell cohorts with spatial transcriptomics, multiplex immunofluorescence, or CITE-seq will be necessary to define how these suppressive myeloid populations are spatially organized relative to *EFNB2*-positive malignant cells and which stromal and immune subpopulations contribute most strongly to the high-risk state.

A key strength of this study is the identification of *EFNB2* as a candidate molecular hub linking histomorphologic phenotype, risk state, and neuron–tumor interaction. *EFNB2* was significantly associated with worse survival in the overall cohort, was markedly enriched in glioblastoma, and showed the highest continuous expression level in *IDH*-wildtype GBM across WHO-defined glioma subtypes. Previous work has implicated the EFNB2/Eph receptor axis in glioma progression. Phosphorylation of ephrin-B2 promotes glioma cell migration and invasion [24]. EFNB2 and its receptor EphB4 are increasingly expressed with higher glioma grade and are associated with poor prognosis in GBM patients [25]. In addition, high *EFNB2* expression and low methylation are associated with adverse outcome in GBM [26]. However, Teng et al. found that EPHB1 ligand-dependent signaling suppresses glioma invasion and correlates with improved patient survival [27]. Recently, Piffko et al. emphasized that ephrinB2-EphB4 signaling has context-dependent and even Janus-faced functions in neuro-oncological disease, depending on cell type, receptor–ligand pairing, and microenvironmental context [28]. This interpretation fits well with our findings. Although *EFNB2* was prognostically significant in the entire cohort, its prognostic effect disappeared within the GBM and *IDH*-mutant subgroups, indicating that *EFNB2* may primarily represent a GBM-enriched malignant cell state rather than a universal subtype-independent prognostic gene. This point is particularly important because it shifts the interpretation of *EFNB2* away from that of a simple single-gene prognostic biomarker. Instead, *EFNB2* appears to be a marker of a specific malignant ecological state closely coupled to glioblastoma biology. In this context, its value lies less in universal prognostic prediction and more in its ability to connect histomorphologic phenotype, WHO-defined subtype composition, and a candidate mechanism of neural interaction. Therefore, the translational value of *EFNB2* at the current stage lies mainly in mechanistic positioning and candidate pathway prioritization, rather than immediate use as a universal prognostic biomarker or therapeutic target. Given that Eph/ephrin signaling is fundamentally involved in developmental processes such as axon guidance and tissue patterning, and also in tumor invasion, angiogenesis, and cell–cell signaling [24,26,27,28], it is biologically plausible that *EFNB2* occupies a meaningful position at the neuron–tumor interface.

The implication of *EFNB2* being identified as a key hub gene is not merely that it is enriched in glioblastoma, but that it appears to represent a biologically informative communication node linking histomorphology, high-risk state, and neuron–tumor interaction. *EFNB2* emerged from the intersection of the synaptogenesis-associated histomorphologic signature, transcriptomic differential analysis, and single-nucleus communication analysis. At the single-cell level, *EFNB2*-positive malignant cells showed the highest node strength in the inferred communication network, and the EFNB2/EPHB1 axis was repeatedly identified between *EFNB2*-positive malignant cells and neurons. Together with the enrichment of additional synapse-related signaling pairs, these findings suggest that *EFNB2* may mark a malignant cell state that is particularly active in neuron-related communication and capable of translating neuronal inputs into tumor-promoting programs.

From a translational perspective, the EFNB2/EPHB1 axis may represent a candidate therapeutic pathway through which *EFNB2*-positive malignant cells engage in neuron–tumor communication. In principle, therapeutic intervention could be envisioned at multiple levels, including disruption of ligand/receptor interactions, attenuation of downstream signaling consequences, or combination with existing treatment strategies to suppress communication-driven tumor-promoting effects. However, such implications should be interpreted cautiously. Eph/ephrin signaling is highly context-dependent, and its biological effects may vary according to cell type, receptor–ligand pairing, and microenvironmental context. Moreover, because this signaling system also participates in normal developmental and neural processes, therapeutic targeting may face specificity and safety challenges. In addition, the marked intertumoral and intratumoral heterogeneity of glioblastoma suggests that dependency on this axis is unlikely to be uniform across all tumors. Therefore, the EFNB2/EPHB1 pathway should currently be regarded as a context-dependent candidate therapeutic axis rather than an immediately actionable target, and its translational relevance remains to be established through direct functional validation.

The single-nucleus analyses provide more direct cellular support for this hypothesis. *EFNB2* was expressed in malignant cells, astrocytes, neurons, and endothelial cells, but *EFNB2*-positive malignant cells displayed the highest node strength in the functionally filtered communication network, indicating a central role in microenvironmental signaling. Importantly, *EFNB2*-positive and *EFNB2*-negative malignant cells were not merely the same malignant population with different *EFNB2* expression levels; rather, they represented distinct functional states. *EFNB2*-positive malignant cells were more strongly enriched in pathways related to cell cycle, Ras/Rap1 signaling, focal adhesion, adherens junction, protein processing in the endoplasmic reticulum, and complement and coagulation cascades, all of which are associated with growth, migration, stress adaptation, and microenvironmental remodeling. In contrast, neurons were enriched in glutamatergic synapse, dopaminergic synapse, and long-term potentiation, consistent with a role in neural transmission and synaptic plasticity. Taken together with prior work showing that neuronal activity drives glioma growth through NLGN3 [19], synaptic integration [13,14], tumor networking [20], and human circuit remodeling [21], these findings support a model in which neurons provide activity- and synapse-related input, whereas *EFNB2*-positive malignant cells may be particularly well positioned to translate such neural cues into pro-tumor programs.

Of particular interest, the ligand-receptor analyses repeatedly identified the EFNB2-EPHB1 axis when *EFNB2*-positive malignant cells and neurons were analyzed as either upstream or downstream clusters. In addition, several signaling pairs closely related to neural development and synaptic function—such as NLGN1/NRXN1, NLGN1/NRXN3, NRG1/ERBB4, NRG3/ERBB4, NXPH1/NRXN1, and NXPH1/NRXN3—were also enriched in the communication network linking *EFNB2*-positive malignant cells and neurons. These observations suggest that the signaling state represented by *EFNB2*-positive malignant cells is not simply a generic malignant–microenvironment interaction pattern, but rather one with developmental-like or synapse-like properties.

SCENIC analysis further supports the idea that *EFNB2*-positive malignant cells are defined by a broader regulatory state, rather than by expression of a single marker gene. It is important to note that the SCENIC heatmap reflects regulon activity, not average gene expression. In our data, JUN, JUND, STAT1, STAT2, STAT3, FOXO3, EP300, RUNX1, and ELK4 regulons were more active in *EFNB2*-positive malignant cells, whereas SOX4, SOX11, TCF4, PBX3, and related regulons were relatively more active in *EFNB2*-negative malignant cells. Although these regulatory patterns require functional validation, they suggest that *EFNB2*-positive malignant cells are characterized by a program combining inflammatory signaling, stress adaptation, cellular activation, and tumor-associated remodeling, rather than merely reflecting lineage identity. Such a regulatory landscape is consistent with the enhanced communication activity and tumor-promoting pathway enrichment observed in these cells.

The value of integrating H&E histomorphology, transcriptomic programs, and clinical outcome data lies in the fact that these three layers contribute complementary information to survival modeling. Histomorphology provides clinically accessible tissue-level information, including nuclear morphology, tissue architecture, and microenvironment-related patterns, but is also highly complex and potentially noisy when used alone. Transcriptomic data in our framework did not serve merely as an additional predictive modality; rather, they acted as a biological anchor that enabled us to identify histomorphologic features specifically linked to synaptogenesis-related molecular activity. Clinical follow-up data then provided the prognostic endpoint needed to refine these biologically informed features into a survival-relevant risk model. Therefore, this integrative strategy improved not only predictive robustness but also interpretability, by linking the final pathology-derived risk score to a defined biological program and validated clinical outcomes.

Several limitations should be acknowledged. First, the PRS was developed and validated within the TCGA framework, and its generalizability remains to be tested in independent external whole-slide image cohorts. Second, the present study provides primarily mechanistic clues rather than causal proof: the proposed role of the EFNB2-EPHB1 axis and the central position of *EFNB2*-positive malignant cells are supported by correlation, cross-platform consistency, and ligand–receptor inference, but still require direct validation through neuron–tumor co-culture systems, electrophysiological assays, spatial profiling, and genetic perturbation experiments. Third, although ESTIMATE and bulk transcriptomic analyses suggested substantial microenvironmental remodeling in the high-risk group, these approaches remain relatively coarse. To partially address this issue, we performed an additional exploratory single-nucleus analysis focused on the myeloid compartment, which provided preliminary cellular support for a shift toward suppressive TAM-like states in GBM. However, this analysis was based on a limited number of samples and was restricted to myeloid cells; therefore, it does not fully resolve how malignant, immune, and stromal subpopulations collectively contribute to the histomorphologic signature, nor does it define their spatial organization within the tumor ecosystem. Consequently, the current study remains limited in its ability to determine whether the biological and prognostic significance of the PRS varies across all cellular compartments or to make robust cell-type-specific therapeutic predictions. Recent studies in other tumor types have similarly underscored that integrating cellular heterogeneity with immunophenotyping, as well as resolving tumor microenvironment heterogeneity, is important for improving biological interpretation and treatment-response prediction [29,30]. Future studies incorporating larger single-cell cohorts together with spatial transcriptomics, multiplex immunofluorescence, CITE-seq, or other spatially resolved multi-omic approaches will be necessary to define the precise cellular composition, functional states, and spatial interactions underlying the *EFNB2*-associated microenvironment and the histomorphologic signature.

## 4. Materials and Methods

### 4.1. Data Source and Preprocessing

Histopathological WSIs of glioma were obtained from the Genomic Data Commons (GDC) portal (https://portal.gdc.cancer.gov/, accessed on 1 November 2025). WSI preprocessing included identification of usable tissue regions and generation of image tiles for downstream analysis. Briefly, 10 non-overlapping image tiles (512 × 512 pixels) were extracted from each WSI. Tiles were excluded if tissue coverage was <75%. To ensure image quality, all candidate tiles were jointly reviewed by two clinicians and two pathologists, and low-quality tiles were removed if they contained bubbles, blur, handwriting marks, tissue folds, or excessive blank background (Figure 1A–C).

Transcriptomic and clinical data for TCGA glioma, including RNA-seq expression profiles from both GBM and lower-grade glioma (LGG), as well as OS and progression-free survival (PFS) data, were obtained from the UCSC Xena platform (https://xena.ucsc.edu/, accessed on 29 December 2022). Molecular alteration data were collected from a previously published study [6]. In addition, the updated 2021 WHO CNS classification information matched to the TCGA glioma transcriptomic samples was curated from another published study [31] and used for subsequent subtype-based analyses. Single-nucleus transcriptomic data used for validation were obtained from two previously published datasets. The GBM dataset was downloaded from Gene Expression Omnibus (GSE274987) [32], whereas the astrocytoma grade 2 dataset was obtained from Zenodo (https://zenodo.org/records/10435521, accessed on 4 October 2025).

The gene set used in this study consisted of synaptogenesis-related genes, which was curated through a systematic summary of previously published studies [33,34,35,36,37,38,39,40,41,42,43,44,45,46].

To further validate the expression patterns of candidate genes across glioma grades at the protein level, immunohistochemical staining data were retrieved from the HPA database. Representative staining images were reviewed to assess expression trends of the target genes across gliomas of different WHO-defined grades.

### 4.2. Feature Extraction from Histopathological Tiles

After WSI preprocessing and selection of high-quality tiles, histomorphologic feature extraction was performed for each tile. To comprehensively characterize glioma histomorphology, we extracted both 2048 ResNet50-derived deep learning features and 630 CellProfiler-derived quantitative histomorphologic features. For deep learning-based feature extraction, a pretrained ResNet50 network was used to encode each tile, generating a 2048-dimensional feature vector. These tile-level deep learning features were then averaged across all tiles from the same case. For quantitative histomorphologic feature extraction, each tile was analyzed using CellProfiler (version 4.2.4). Briefly, hematoxylin and eosin staining signals were used to identify and segment nuclei and tissue regions, followed by extraction of multiple interpretable histomorphologic features, including intensity, texture, granularity, size and shape descriptors, object adjacency measurements, and nuclear morphology-related metrics. At the case level, the mean, median, and standard deviation of each CellProfiler-derived feature across all tiles were calculated to summarize both the overall level and spatial heterogeneity of the histomorphologic characteristics. Together, the 2048 ResNet50-derived deep learning features and 630 CellProfiler-derived quantitative histomorphologic features constituted an integrated histomorphologic feature set of 2678 features for downstream analyses.

### 4.3. Histomorphologic Feature Selection and Association with Synaptogenesis-Related Gene Programs

To link histomorphologic features with synaptogenesis-related molecular activity, we first quantified the activity of a synaptogenesis-related gene set in the TCGA glioma transcriptomic dataset using ssGSEA implemented in the GSVA package (Version 2.0.7). This approach generated a sample-wise enrichment score for each tumor, providing a quantitative estimate of synaptogenesis-related biological activity at the transcriptomic level. The resulting gene set scores were then correlated with the case-level integrated histomorphologic feature set. For each histomorphologic feature, Spearman’s rank correlation analysis was performed to assess its association with the synaptogenesis-related enrichment score, and features showing significant correlations were retained as candidate histomorphologic features. To further refine the feature set and reduce redundancy, the candidate features were subjected to elastic net regularization using the glmnet package (Version 4.1-10) with α = 0.2. This procedure allowed us to retain a compact set of representative and robust histomorphologic features associated with synaptogenesis-related gene programs for subsequent survival modeling and biological interpretation.

### 4.4. Prognostic Feature Selection and Machine Learning Model Construction

After identifying histomorphologic features associated with synaptogenesis-related gene programs, the features retained by elastic net regularization (glmnet, α = 0.2) were further subjected to univariate Cox proportional hazards regression analysis to identify candidate variables significantly associated with patient survival. Only histomorphologic features showing statistical significance in the univariate analysis, together with the corresponding survival information, were then incorporated into the MIME1 framework to develop machine learning-based survival models. C-index was used as the primary performance metric to evaluate model discrimination, and the model with the best overall performance was selected as the final prognostic model.

After the optimal model was selected, a risk score was calculated for each sample according to the corresponding model coefficients. Patients were then stratified into high-risk and low-risk groups using the median PRS as the cutoff. Kaplan–Meier survival curves were generated to compare survival outcomes between the two groups, and differences were assessed using the log-rank test. In parallel, for key genes of interest, patients were also divided into high-expression and low-expression groups based on the median expression level, followed by survival analysis using the same approach. To further assess time-dependent predictive performance, the AUC values for 1-, 3-, and 5-year survival were calculated, and time-dependent ROC curves were plotted.

To determine whether PRS could serve as an independent predictor of patient survival, both univariate and multivariate Cox regression analyses were performed. Univariate analysis was used to evaluate the association between each clinicopathological variable, including PRS, and survival outcome. Multivariate analysis was then conducted by incorporating PRS together with other major clinical covariates to determine whether it retained prognostic significance after adjustment for potential confounding factors.

### 4.5. Differential Expression Analysis, Functional Enrichment Analysis, and Immune Microenvironment Assessment Between Risk Groups

To investigate the molecular differences associated with distinct risk states, patients were stratified into high-risk and low-risk groups according to PRS. Differential gene expression analysis between the two groups was then performed using the limma package (Version 3.62.2). Significantly differentially expressed genes were identified to characterize the molecular alterations associated with risk stratification.

After identifying differentially expressed genes between the high- and low-risk groups, functional enrichment analysis was conducted to clarify the biological processes and signaling pathways represented by these genes. Specifically, both Gene Ontology (GO) enrichment analysis and KEGG pathway analysis were performed to identify key biological functions, molecular mechanisms, and pathway alterations associated with the risk phenotype.

To assess differences in tumor microenvironment composition between the two risk groups, the estimate package (Version 1.0.13) was applied to the TCGA glioma transcriptomic dataset. This method was used to calculate the ImmuneScore, StromalScore, and ESTIMATEScore, which reflect the relative abundance of immune components, stromal components, and overall non-tumor microenvironmental infiltration within each sample. These scores were then compared between the high-risk and low-risk groups to evaluate the relationship between PRS and the immune microenvironmental landscape.

### 4.6. Correlation Analysis Between Histomorphologic Features and Synaptogenesis-Related Genes and Identification of the Hub Gene

To further connect histomorphologic features with synaptogenesis-related molecular characteristics, correlation analysis was performed between the selected key histomorphologic features and the expression levels of synaptogenesis-related genes. Based on the TCGA glioma transcriptomic dataset, the expression matrix of synaptogenesis-related genes was extracted and matched to the case-level histomorphologic feature matrix. Spearman’s rank correlation analysis was then used to evaluate the strength of association between each histomorphologic feature and each synaptogenesis-related gene. The resulting correlations were used to generate a histomorphologic feature–gene association matrix and served as the basis for subsequent hub gene identification.

To identify key molecules associated with both histomorphologic features and risk stratification, we intersected the candidate genes derived from the histomorphologic feature-synaptogenesis gene correlation analysis with the significantly differentially expressed genes identified between the high- and low-risk groups. Among the overlapping genes, candidates were ranked according to the adjusted *p* values from the differential expression analysis, and the gene with the smallest adjusted *p* value was defined as the hub gene. This gene was considered the most representative candidate linking histomorphologic, synaptogenesis-related molecular programs, and prognostic risk status, and was selected for downstream survival analysis, biological validation, and mechanistic investigation.

### 4.7. snRNA-seq Data Analysis

The overall workflow for snRNA-seq data processing is summarized in Supplementary Figure S4. Cell types were manually annotated according to the expression patterns of canonical marker genes. The marker genes used for annotation were collected from multiple previously published studies and interpreted in conjunction with their expression distributions in the present dataset (Supplementary Figures S5 and S6). To investigate intercellular signaling interactions among different cell populations, CommPath was used for cell–cell communication analysis. This method infers potential signaling relationships based on ligand–receptor expression patterns and further evaluates the relative communication strength of specific cell populations within the global interaction network. To further characterize the transcriptional regulatory programs underlying *EFNB2*-associated malignant cell states, SCENIC was performed for transcription factor network inference. Given the substantial computational demand of this analysis, 2000 cells were randomly sampled from the integrated snRNA-seq dataset for SCENIC analysis. This approach was used to identify regulons composed of transcription factors and their putative target genes and to compare regulon activity across different cell populations, thereby providing insight into the potential upstream regulatory mechanisms associated with *EFNB2*-related cellular states.

### 4.8. Exploratory Re-Analysis of the Myeloid Compartment

To further investigate myeloid heterogeneity in relation to glioma grade and *EFNB2*-associated malignant context, we performed an exploratory re-analysis of the myeloid compartment in the integrated snRNA-seq dataset. Cells annotated as myeloid cells were subset from the integrated object and reprocessed, including normalization, identification of highly variable genes, scaling, principal component analysis, Harmony-based batch correction using patient identity, graph-based clustering, and UMAP visualization. Clustering resolution was set to 0.4 for the final exploratory analysis. Based on clustering pattern, canonical marker expression, and module scores for *APOE/C1QC*-like, *SPP1*-associated, M2-like, and homeostatic microglial programs, myeloid cells were re-annotated into four major states: Microglia-like, Suppressive TAM-like, Transitional myeloid, and Stress-ambiguous populations. The Suppressive TAM-like population was defined as the major tumor-associated suppressive myeloid state for downstream quantitative analysis. To compare myeloid composition across biological groups, cell-state proportions were calculated at the sample level within the myeloid compartment. Two-group comparisons, including astrocytoma grade 2 versus GBM and *EFNB2*-high versus *EFNB2*-low malignant contexts, were performed using the Wilcoxon rank-sum test at the sample level because of the limited number of independent samples and the non-Gaussian nature of proportion data. For *EFNB2*-context analysis, malignant cells were extracted from the integrated snRNA-seq object, and sample-level *EFNB2* context was defined according to the sample-wise mean *EFNB2* expression in malignant cells, dichotomized by the median value.

## 5. Conclusions

The significance of this study lies not only in the development of a pathology-derived prognostic risk model but also in demonstrating that neural-related molecular programs can be read out from routine H&E morphology. More importantly, this readout points toward specific molecules and intercellular interaction axes that may be mechanistically meaningful. Our findings nominate *EFNB2*-positive malignant cells as a promising candidate hub in glioma neuron–tumor interaction. If future functional studies confirm the role of EFNB2-EPHB1 and related neuronal activity-dependent pathways in glioma progression, this line of investigation may deepen our understanding of glioma biology in the context of cancer neuroscience and open new avenues for risk stratification and therapeutic targeting.

## Data Availability

The data presented in this study are available in the public domain. Histopathological whole-slide images were obtained from the Genomic Data Commons (GDC) portal at https://portal.gdc.cancer.gov/ (accessed on 1 November 2025). Transcriptomic and clinical data for TCGA glioma were obtained from UCSC Xena at https://xena.ucsc.edu/ (accessed on 29 December 2022). The glioblastoma single-nucleus RNA-seq dataset is available in the Gene Expression Omnibus (GEO) at https://www.ncbi.nlm.nih.gov/geo/query/acc.cgi?acc=GSE274987 (accessed on 4 October 2025), reference number GSE274987. The astrocytoma grade 2 single-nucleus RNA-seq dataset is available in Zenodo at https://zenodo.org/records/10435521 (accessed on 4 October 2025), reference number 10435521. Immunohistochemical data were obtained from the Human Protein Atlas at https://www.proteinatlas.org/ (accessed on 31 January 2026). Additional molecular alteration data were derived from Ceccarelli et al. [6], and the updated 2021 WHO CNS classification annotations matched to the TCGA glioma transcriptomic samples were curated from Zakharova et al. [31].

## Supplementary Materials

The following supporting information can be downloaded at: https://www.mdpi.com/article/10.3390/ijms27104300/s1.

## Abbreviations

The following abbreviations are used in this manuscript:

WHO CNS	World Health Organization Classification of Tumors of the Central Nervous System
H&E	Hematoxylin and eosin
WSIs	Whole-slide images
PRS	Pathology-derived risk score
TCGA	The cancer genome atlas
GSVA	Gene Set Variation Analysis
ssGSEA	Single-sample gene set enrichment analysis
HPA	Human Protein Atlas
OS	Overall survival
HR	Hazard ratios
CI	Confidence interval
plsRcox	Partial least squares Cox regression
RSF	Random survival forest
GBM	Gradient boosting machine
Enet	Elastic net
survival-SVM	Survival support vector machine
C-index	Harrell’s concordance index
ROC	Receiver Operating Characteristic
AUC	Area under the curve
KEGG	Kyoto Encyclopedia of Genes and Genomes
GO	Gene Ontology
BP	Biological Process
CC	Cellular Component
MF	Molecular Function
MHC	Major Histocompatibility Complex
CAM	Cell adhesion molecule
LGG	lower-grade glioma
PFS	progression-free survival
GDC	Genomic Data Commons
TAM	Tumor-associated macrophage
