# Supplementary material for: A Synaptogenesis-Associated Histomorphologic Signature from H&E Whole-Slide Images Predicts Glioma Prognosis and Identifies EFNB2-Positive Malignant Cells as a Candidate Neuro-Glioma Communication Hub
Source: Int J Mol Sci. 2026 May 12;27(10):4300. doi: 10.3390/ijms27104300 (PMC13207882; doi:10.3390/ijms27104300)
Supplement: Supplementary file 1 [file ijms-27-04300-s001.zip › Supplementary figure legends.pdf]

Supplementary Figure S1. Cell-cell communication analysis.

Supplementary Figure S2. Exploratory re-annotation of the myeloid compartment in the integrated snRNA-seq dataset. (A) UMAP visualization of re-clustered myeloid cells at resolution = 0.4, shown separately for astrocytoma grade 2 and glioblastoma (GBM). (B) Dot plot of representative marker genes used for myeloid subcluster annotation, including homeostatic microglial markers and tumor-associated/suppressive myeloid markers. Dot size indicates the proportion of expressing cells, and color indicates average expression level. (C) UMAP visualization of the final re-annotated myeloid states, including Microglia-like, Suppressive TAM-like, Transitional myeloid, and Stress-ambiguous populations, shown separately for astrocytoma grade 2 and GBM. The astrocytoma grade 2 was dominated by Microglia-like cells, whereas GBM was enriched for Suppressive TAM-like cells.

Supplementary Figure S3. Suppressive myeloid cell proportion in EFNB2-associated malignant contexts. Sample-level proportions of suppressive myeloid cells within the myeloid compartment were compared between *EFNB2*-high and *EFNB2*-low malignant contexts. Samples with higher malignant-cell *EFNB2* expression showed a directional trend toward increased suppressive myeloid proportions, although the difference did not reach statistical significance (Wilcoxon $P = 0.144$).

Supplementary Figure S4. Flow chart of processing snRNA-seq data.

Supplementary Figure S5. Quality control and screening highly variable genes from snRNA-seq data. (A) Bar chart of the number of genes per cell (nFeature_RNA), the number of UMIs per cell (nCount_RNA), and the proportion of intracellular mitochondrial gene expression (percent.mt). (B) Dot diagram of the average expression of top 4,000 highly variable genes in red (Top 10 highly variable genes are labeled independently). UMIs, unique molecular identifers.

Supplementary Figure S6. Dimensionality reduction clustering analysis and cell annotation of marker genes in each cell cluster. (A) UMAP plot of identification of 24 cell clusters. (B) Dot plot of the expression level of marker genes in cell clusters. The size of the dot represented the percentage of expression level of genes in each cluster, and the color represented the average expression level of genes by log normalized methods. UMAP, performing uniform manifold approximation and projection.
