# Supplementary material for: A Synaptogenesis-Associated Histomorphologic Signature from H&E Whole-Slide Images Predicts Glioma Prognosis and Identifies EFNB2-Positive Malignant Cells as a Candidate Neuro-Glioma Communication Hub
Source: Int J Mol Sci. 2026 May 12;27(10):4300. doi: 10.3390/ijms27104300 (PMC13207882; doi:10.3390/ijms27104300)
Supplement: Supplementary file 1 [file ijms-27-04300-s001.zip › supplementary tables.pdf]

Supplementary Table S1. Synaptogenesis-related genes

RTN4R	PTPRS	C1QL2	CA10	MDGA2	SLITRK1
RTN4RL1	NTNG1	C1QL3	CA11	NLGN1	SLITRK3
RTN4RL2	NTNG2	C1QL4	TAFA1	NLGN2	SLITRK4
TENM1	NPTX1	NTN1	TAFA2	NLGN3	SLITRK5
TENM2	NPTX2	NTN3	TAFA3	NLGN4X	SLITRK6
TENM3	NPTXR	NTN4	TAFA4	NLGN4Y	IL1RAP
TENM4	CADM1	NTN5	ADGRB1	DAG1	LRRC4B
FLRT1	CADM2	CBLN1	ADGRB2	LRRTM1	LRRC4C
FLRT2	EPHB1	CBLN2	ADGRB3	LRRTM2	LRRC4
FLRT3	EPHB2	CBLN3	UNC5A	LRRTM3	GRIA1
NRXN1	EPHB3	CBLN4	UNC5B	LRRTM4	GRIA2
NRXN2	EPHB4	NXPH1	UNC5C	CLSTN2	GRIA3
NRXN3	CDH8	NXPH2	UNC5D	CLSTN1	GRIA4
PTPRD	CDH9	NXPH3	GLUD1	CLSTN3	EFNB1
PTPRF	C1QL1	NXPH4	MDGA1	NTRK3	EFNB2
					EFNB3

Supplementary Table S2. Sample information single nucleus RNA-sequencing data

Sample ID	WHO2021	Sample ID in the original literature
Sample 1	Astrocytoma, Grade 2	110_R1
Sample 2	Astrocytoma, Grade 2	115_R1
Sample 3	Astrocytoma, Grade 2	131_R2
Sample 4	Astrocytoma, Grade 2	113_R1
Sample 5	Glioblastoma, Grade 4	6419_D
Sample 6	Glioblastoma, Grade 4	6425_E
Sample 7	Glioblastoma, Grade 4	6434_A
Sample 8	Glioblastoma, Grade 4	6467_E
Sample 9	Glioblastoma, Grade 4	6509_D
Sample 10	Glioblastoma, Grade 4	6514_B

Supplementary Table S3. Fourteen histomorphologic features with non-zero coefficients retained Enet[0.1] model

Feature	Source	Coefficient	Suggested interpretation
Total.Granularity.3.Hematoxylin	CellProfiler	0.1342	Measures coarse-grained texture patterns in the hematoxylin channel, likely reflecting increased nuclear/chromatin granularity and staining heterogeneity.
Total.Texture.Correlation.Hematoxylin.3.01.256	CellProfiler	-0.0105	A texture correlation metric in the hematoxylin channel reflecting the spatial dependency and regularity of staining intensity; may relate to chromatin organization smoothness.
Object.Mean.IdentifyPrimaryObjects.AreaShape.Eccentricity	CellProfiler	0.1895	Mean eccentricity of segmented primary objects (predominantly nuclei); higher values suggest more elongated or anisotropic nuclear morphology.
Object.Mean.IdentifyPrimaryObjects.AreaShape.Zernike.2.0	CellProfiler	0.2334	A low-order Zernike shape descriptor capturing global nuclear geometry and coarse shape symmetry.
Object.Median.IdentifyPrimaryObjects.AreaShape.Eccentricity	CellProfiler	0.1812	Median eccentricity of segmented nuclei; reflects the central tendency of nuclear elongation across the tissue tile.
Object.Median.IdentifyPrimaryObjects.AreaShape.Zernike.5.5	CellProfiler	-0.0427	A higher-order Zernike moment reflecting more subtle nuclear contour characteristics and shape complexity.
Object.Median.IdentifyPrimaryObjects.AreaShape.Zernike.7.3	CellProfiler	-0.0248	A higher-order nuclear shape descriptor that may reflect fine-scale contour irregularity and geometric heterogeneity.
Object.Median.IdentifyPrimaryObjects.AreaShape.Zernike.7.7	CellProfiler	-0.05	A higher-order Zernike feature related to detailed nuclear contour geometry and symmetry deviation.
Object.Median.IdentifyPrimaryObjects.AreaShape.	CellProfiler	-0.0297	A high-order shape descriptor capturing complex nuclear

Zernike.9.3			boundary patterns.
Object.StDev.IdentifyPrimaryObjects.Texture.Correlation.Hematoxylin.3.00.256	CellProfiler	-0.0101	Standard deviation of nuclear texture correlation across segmented objects, reflecting intercellular variability in chromatin/staining organization.
resnet429	ResNet50	0.1519	Deep latent feature automatically learned from image patches; likely captures higher-order morphologic information not directly summarized by handcrafted descriptors.
resnet1061	ResNet50	-0.2075	Deep latent feature potentially reflecting composite tissue architectural and textural patterns; exact morphologic meaning is not directly interpretable.
resnet1163	ResNet50	0.0593	Deep latent feature representing abstract visual patterns learned by the network, possibly related to tissue organization and cellular arrangement.
resnet1573	ResNet50	0.1506	Deep latent feature likely encoding integrated high-level morphologic signals, such as microarchitectural heterogeneity and texture complexity.

Suggested interpretations were derived from the formal definitions of the corresponding image features and are provided as putative histomorphologic explanations to improve interpretability; they should not be regarded as direct biological annotations.
