# Supplementary figures and images for: A Synaptogenesis-Associated Histomorphologic Signature from H&E Whole-Slide Images Predicts Glioma Prognosis and Identifies EFNB2-Positive Malignant Cells as a Candidate Neuro-Glioma Communication Hub
Source: Int J Mol Sci. 2026 May 12;27(10):4300. doi: 10.3390/ijms27104300 (PMC13207882; doi:10.3390/ijms27104300)

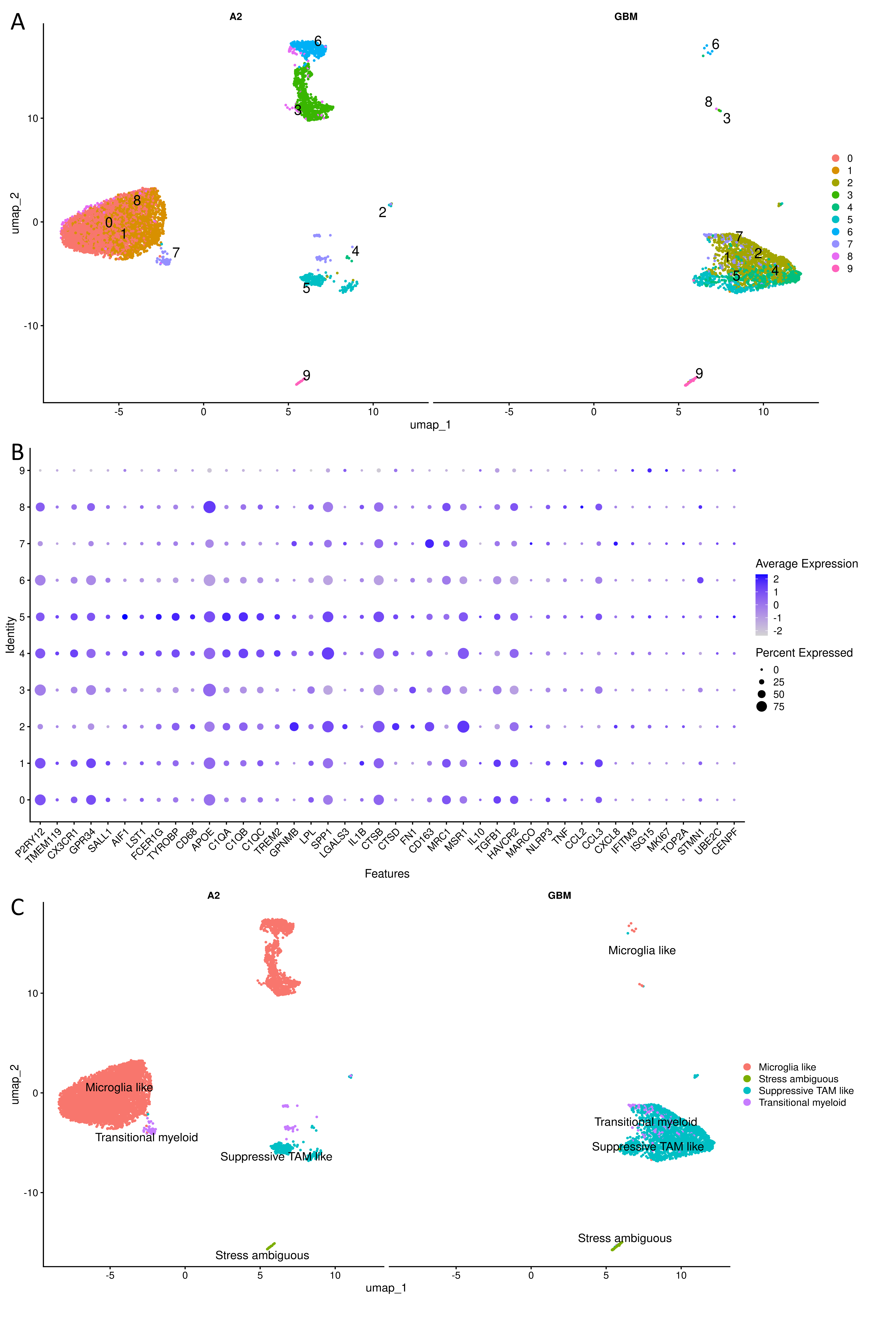

Supplement: Supplementary file 1 [file ijms-27-04300-s001.zip › Supplementary Figure S2.png]

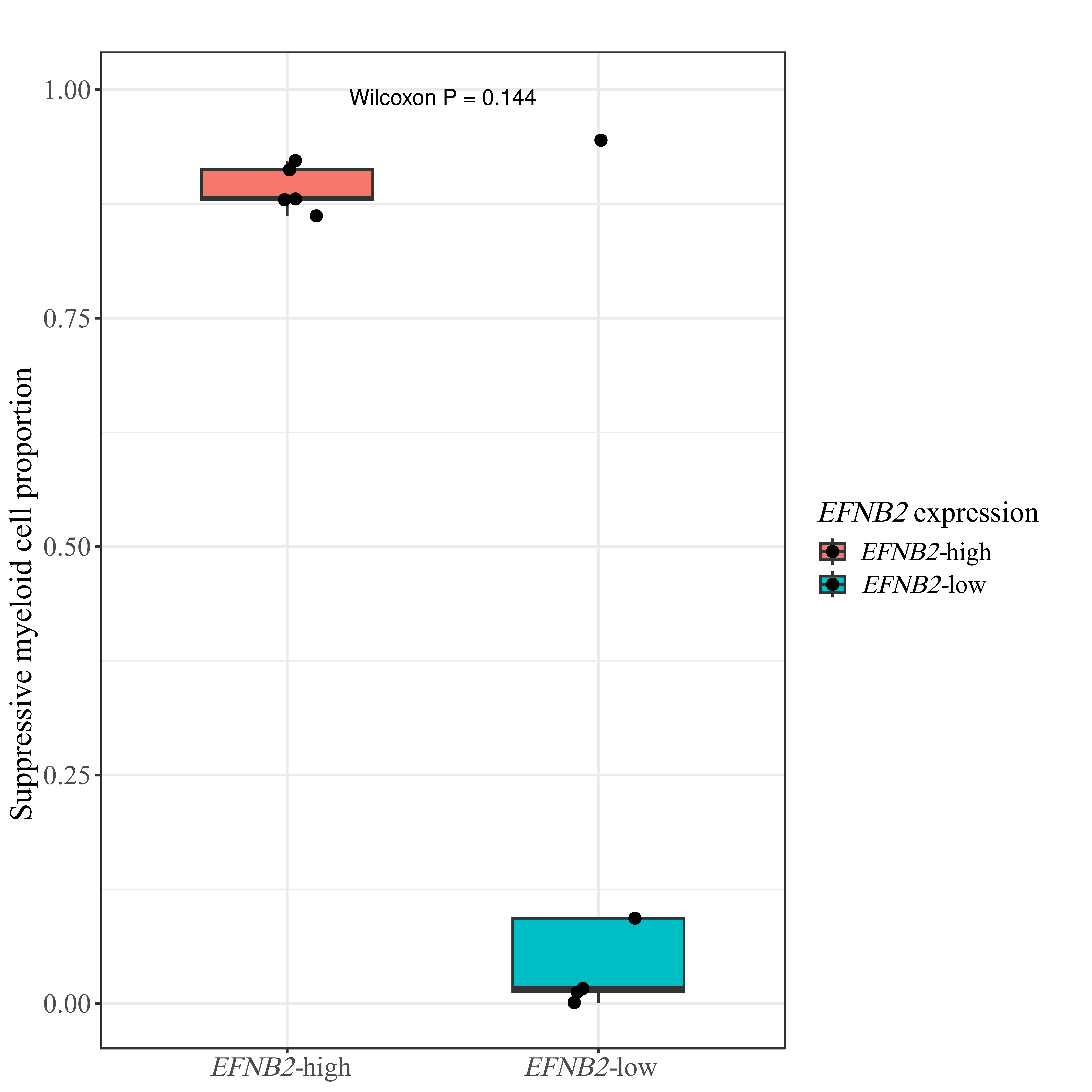

Supplement: Supplementary file 1 [file ijms-27-04300-s001.zip › Supplementary Figure S3.png]

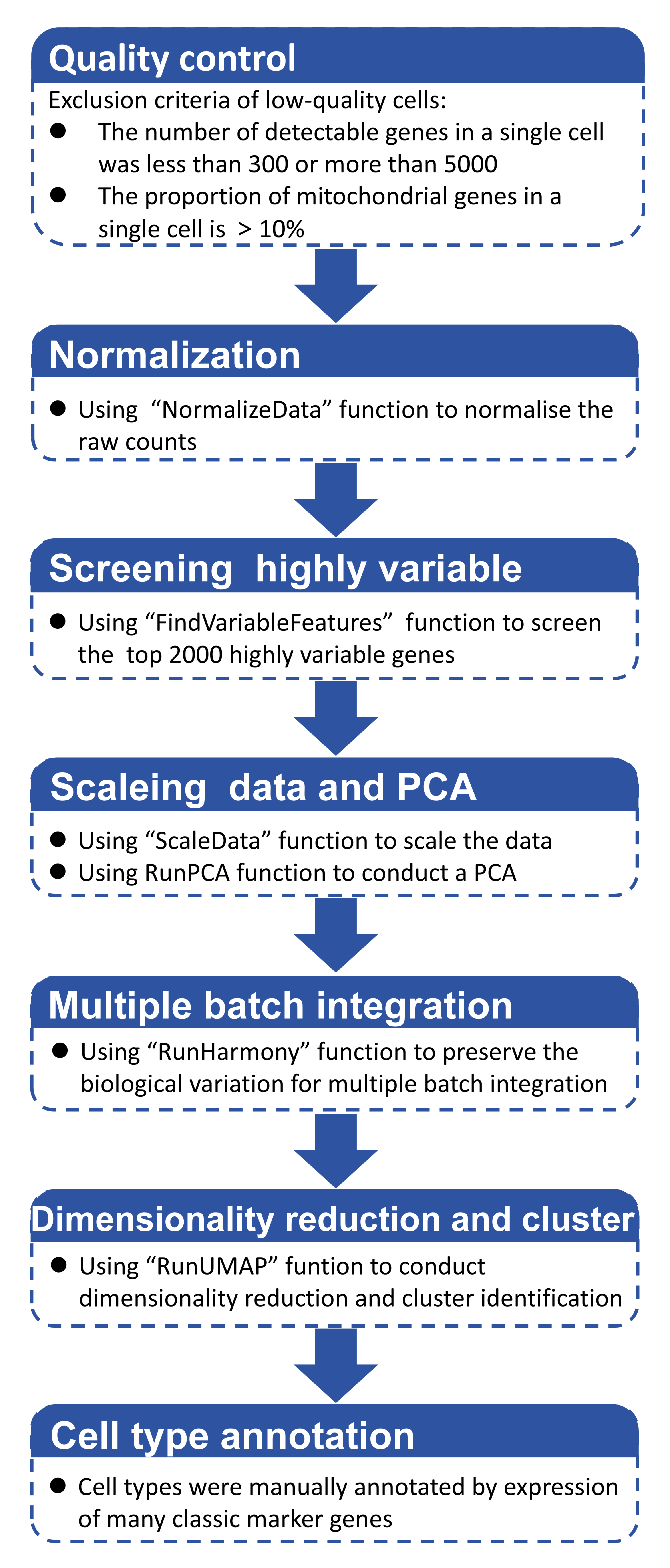

Supplement: Supplementary file 1 [file ijms-27-04300-s001.zip › Supplementary Figure S4.png]

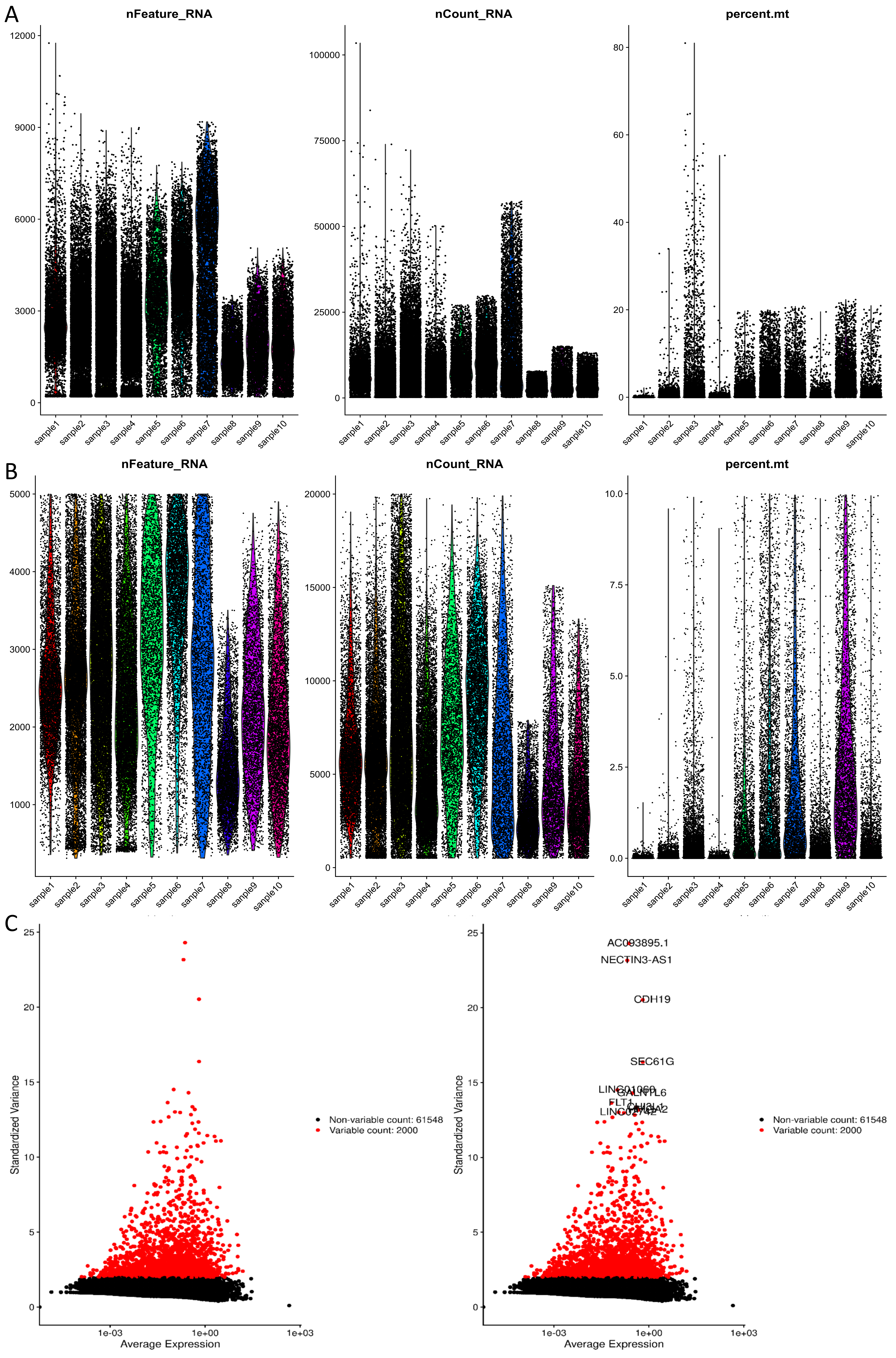

Supplement: Supplementary file 1 [file ijms-27-04300-s001.zip › Supplementary Figure S5.png]

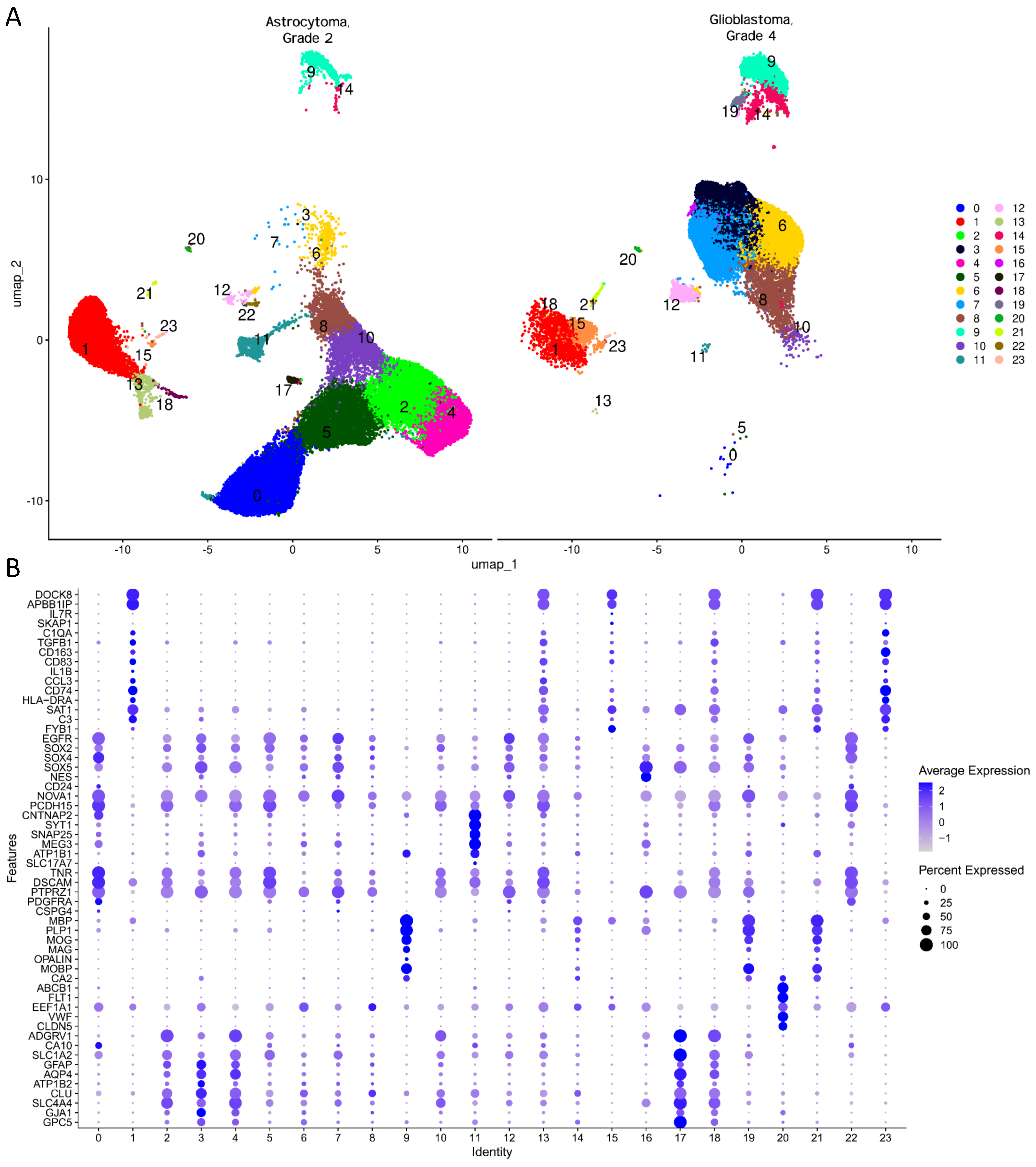

Supplement: Supplementary file 1 [file ijms-27-04300-s001.zip › Supplementary Figure S6.png]
